# Neurocognitive functioning in patients with conversion disorder/functional neurological disorder

**DOI:** 10.1111/jnp.12206

**Published:** 2020-03-29

**Authors:** Lars de Vroege, Iris Koppenol, Willem Johan Kop, Madelon M.E. Riem, Christina Maria van der Feltz‐Cornelis

**Affiliations:** ^1^ Clinical Centre of Excellence for Body, Mind, and Health GGz Breburg Tilburg the Netherlands; ^2^ Tilburg School of Social and Behavioral Sciences Tranzo Department Tilburg University the Netherlands; ^3^ Department of Medical and Clinical Psychology Center of Research on Psychological and Somatic disorders (CoRPS) Tilburg University the Netherlands; ^4^ Mental Health and Addiction Research Group (MHARG) Department of Health Sciences HYMS University of York UK

**Keywords:** conversion disorder, functional neurological disorder, neurocognitive functioning, neuropsychology, somatic symptom and related disorders

## Abstract

Neurocognitive symptoms are common in individuals with somatic symptom and related disorders (SSRD), but little is known about the specific impairments in neurocognitive domains in patients with conversion disorder (CD)/functional neurological disorder (FND). This study examines neurocognitive functioning in patients with CD/FND compared to patients with other SSRD. The sample consisted of 318 patients. Twenty‐nine patients were diagnosed with CD/FND, mean age 42.4, *standard deviation (SD)* = 13.8 years, 79.3% women, and 289 patients had other SSRD (mean age 42.1, *SD* = 13.3, 60.2% women). Patients completed a neuropsychological test battery that addressed a broad range of neurocognitive domains, including information processing speed, attention and executive functioning. Patients with CD/FND had clinically significant neurocognitive deficits in all neurocognitive domains based on normative data comparison. Patients with CD/FND also performed significantly worse than patients with other SSRD on information processing speed (Digit Symbol Substitution Test (*V* = .115, *p* = .035), Stroop Color–Word Test (SCWT) card 1 (*V* = .190, *p* = .006), and SCWT card 2 (*V* = .244, *p* < .001). No CD/FND vs. other SSRD differences were observed in other neurocognitive domains. These findings indicate the patients with CD/FND perform worse on information processing speed tests compared to patients with other SSRD.

## Background

Conversion disorder (CD)/functional neurological disorder (FND) as defined in the DSM‐5 is characterized by the presence of one or more deficits in voluntary motor or sensory functions, which causes significant suffering and a burden of disease in multiple areas of daily life. Additionally, these symptoms cannot be explained by a neurological or other medical condition (American Psychiatric Association (APA), 2013). Patients can display symptoms of motor weakness, abnormal muscle contractions, loss of sensory functions, and/or non‐epileptic seizures (Kozlowska *et al.*, 2015; Krem, 2004). The Diagnostic and Statistical Manual of Mental Disorders – fifth edition (DSM‐5) (APA, 2013) classifies CD/FND among the somatic symptom and related disorders (SSRD) (APA, 2013). Other categories of SSRD are somatic symptom disorder, illness anxiety disorder, factitious disorder, psychological factors affecting other medical conditions, and other somatic symptom and related disorders (APA, 2013).

The prevalence of CD/FND is approximately 0.7–5.0% (Uijen & Bischoff, 2011) and is more common among women than men (Feinstein, 2011; Krem, 2004; Uijen & Bischoff, 2011) with a typical onset between the ages of 10–35 (Uijen & Bischoff, 2011). Risk factors for CD/FND are depression, psychological or physical trauma, and low education (Uijen & Bischoff, 2011). A common factor concerning demographic correlates of adult patients with CD/FND is a lower level and/or fewer years of education (Binzer, Andersen, & Kullgren, 1997; Deka, Chaudhury, Bora, & Kalita, 2007; Kuloglu, Atmaca, Tezcan, Gecici, & Bulut, 2003; Kuwabara *et al.*, 2007; Templer & Lester, 1974), compared to either healthy individuals or people with other SSRD. Psychiatric comorbidity is frequently present in CD/FND, including depression, pain disorders, and personality disorders (Binzer, Andersen & Kullgren, 1997; Krem, 2004). Furthermore, physical stressors (e.g., injury to the concerned limb) also seem to function as a trigger in CD/FND (Stone, Warlow, & Sharpe, 2012). Although conflicts and stressors often contribute to the development and persistence of CD/FND (Feinstein, 2011; Nicholson *et al.*, 2016), there is substantial variability in the factors contributing to the aetiology of CD/FND (Ludwig *et al.*, 2018).

Recent studies have shown that CD/FND is associated with neurocognitive impairments in several domains. Deficits and impairments have been documented in attention (Brown, Nicholson, Aybek, Kanaan, & David, 2014; Demir, Celikel, Taycan, & Etikan, 2013; Kozlowska *et al.*, 2015), learning (Demir *et al.*, 2013), auditory–verbal memory (Brown *et al.*, 2014), (working) memory (Brown *et al.*, 2014; Demir *et al.*, 2013; Kozlowska *et al.*, 2015), executive functioning (Brown *et al.*, 2014; Demir *et al.*, 2013; Kozlowska *et al.*, 2015), and visuospatial functioning (Demir *et al.*, 2013). A limitation of these studies concerns methodological and research design‐related issues. For example, different neuropsychological tests have been used to assess neurocognitive functioning and psychiatric comorbidity has not been taken into account systematically. The results of these studies should therefore be interpreted with caution. Because psychiatric comorbidity is common in CD/FND and other SSRD, particularly depression and anxiety (Binzer *et al.*, 1997; Henningsen, Zimmermann, & Sattel, 2003; Krem, 2004; Van Eck van der Sluijs, Ten Have, Rijnders, Van Marwijk, De Graaf, & Van der Feltz‐Cornelis, 2015), and these comorbid conditions are also accompanied by neurocognitive dysfunction in multiple domains (Castaneda, Tuulio‐Henriksson, Marttunen, Suvisaari, & Lonnqvist, 2008; Lee, Hermens, Porter, & Redoblado‐Hodge, 2012; Rock, Roiser, Riedel, & Blackwell, 2014; Tempesta, Mazza, Serroni, Moschetta, Di Giannantonio, & Ferrara, 2013), these conditions may add to neurocognitive dysfunction in CD/FND and other SSRD (De Vroege, Timmermans, Kop, & Van der Feltz‐Cornelis, 2018).

Research on neurocognitive impairment in patients with CD/FND has typically used normative data or non‐psychiatric (healthy) comparison groups without taking concurrent physical complaints into consideration. It is therefore not known whether the nature and severity of these impairments differ from neurocognitive functioning in patients with other SSRD. For instance, somatization and multiple functional somatic symptoms can cause impairments in semantic memory as well as verbal episodic memory, visuospatial functioning, psychomotor speed, and attention (Hall, Kuzminskyte, Pedersen, Ornbol, & Fink, 2011; Niemi, Porti, Aalto, Hakala, & Karlsson, 2002). Patients with chronic pain also show impairments in attention (Hart, Martelli, & Zasler, 2000; Moore, Keogh, & Eccleston, 2012), processing speed, psychomotor speed, and verbal fluency (Hart, Martelli, & Zasler, 2000). Patients with undifferentiated somatoform disorder are reported to perform poorly on tasks involving working memory and executive functioning (Al‐Adawi, Al‐Zakwani, Obeid, & Zaidan, 2010). Chronic fatigue syndrome can lead to impairments in processing speed, working memory, and information learning (Michiels, & Cluydts, 2001). Patients with fibromyalgia have been found to perform poorly on concentration tests, memory tests, and tests of immediate and delayed recall (Grace *et al.*, 1999). A recent study also concluded that neurocognitive function in patients with SSRD is substantially reduced across a broad array of cognitive domains (De Vroege *et al.*, 2018). These investigations indicate that individuals with SSRD‐alike symptoms and SSRD are characterized by a wide range of neurocognitive impairments and it is not established whether the neurocognitive problems in CD/FND are more profound than in patients with other SSRD.

This background indicates that systematic studies assessing a broad range of neurocognitive domains in patients with CD/FND are limited in number and methodological quality. Furthermore, neurocognitive functioning in CD/FND has not yet been directly compared with neurocognitive functioning in patients with other SSRD. Therefore, this study will examine neurocognitive functioning in individuals with CD/FND and compare them to patients with other SSRD. Considering the clinical features of CD/FND, differences in severity of cognitive dysfunction are expected when comparing to cognitive functioning of patients with other SSRD. We therefore examined neurocognitive functioning in patients with CD/FND and to compare the findings with neurocognitive functioning in patients with other SSRD. It was predicted that patients with CD/FND will have problems in neurocognitive functioning, based on comparison with population‐based normative reference data. Based on earlier studies (Brown *et al.*, 2014; Demir *et al.*, 2013; Kozlowska *et al.*, 2015), these problems are expected especially in the domains attention, (working) memory, planning/executive functioning, and visuospatial functioning. Additionally, we expect that neurocognitive functioning in patients with CD/FND will be poorer compared to patients with other SSRD and that these differences are not accounted for by potential confounders (i.e., age, sex, level of education, depression, and anxiety).

## Methods

### Design

This study used a cross‐sectional design. First, neurocognitive functioning of patients with CD/FND was compared to neurocognitive functioning in the general population normative reference data. Second, neurocognitive functioning of patients with CD/FND was compared to neurocognitive functioning in patients with other SSRD. The neuropsychological assessment (NPA) took place at the clinical centre of Excellence for Body, Mind, and Health (CLGG), a department of a large mental health facility in the Netherlands (GGz Breburg, Tilburg).

Data collection for this study was conducted from September 2013 to April 2017. All patients were informed by letter before intake that their data could be used anonymously for scientific research. Patients could indicate during intake if they declined the use of their data for such purposes. In case patients declined the use of their data, this was recorded in the administration system and the data of these patients were excluded from this study (one patient declined the use of data for scientific research). Not consenting to use of their data had no consequence on treatment at CLGG. The study protocol was approved by the Scientific Review Committee of GGz Breburg (file number: CWO 2019‐05).

### Participants and diagnosis

Participants were consecutive patients presenting with CD/FND or with other SSRD at the CLGG. Inclusion criteria for the study were a diagnosis of either CD/FND or SSRD according to DSM‐5 criteria and the ability to complete the NPA. Patients were excluded if they evidenced malingering based on the tests for malingering, if they had insufficient knowledge of the Dutch language, or if psychotic features or acute suicide risk was present.

Diagnosis of CD/FND and other SSRD was based on clinical evaluation by one of the psychiatrists using DSM‐5 criteria and confirmed in the diagnostic multidisciplinary team discussion. Before intake, referral letters from hospitals and medical specialists were obtained and (re‐) evaluated. These letters included information about electroencephalograms (EEG), computer tomography (CT) scans, and magnetic resonance imaging (MRI) scans that were made previously to obtain information regarding neurological substrates for patients’ neurological symptoms.

### Measures

#### Neuropsychological test battery

The primary outcome of this study is neurocognitive functioning. Patients were classified as either having no neurocognitive problems (larger than or equal to the 20th percentile), having a deficit (between the 2.4th and 20th percentile) or having a disorder (smaller than the 2.4th percentile) (Lezak, Howieson, & Loring, 2012). Results of the neuropsychological tests are categorized in the following domains: information processing speed, attention, divided attention, memory, working memory, language, visuospatial functioning, and executive functioning.

##### Information processing speed

This domain was assessed using the subtest Digit Symbol Substitution Test from the fourth edition of the Wechsler Adult Intelligence Scale (WAIS‐IV; Wechsler, 2008), the Trail Making Test A (TMT‐A; Reitan, 1992), and the Stroop Color–Word Test (SCWT), card 1 and card 2 (Stroop, 1935). The WAIS Digit Symbol Substitution Test examines information processing speed and psychomotor speed by asking patients to fill in the corresponding symbols to the numbers presented on the piece of paper (Wechsler, 2008). In subtest A of the TMT, patients are asked to connect the presented numbers in ascending order (Reitan, 1992). The SCWT consists of three cards (Stroop, 1935). In card 1, patients read aloud a list of the words red, green, blue, and yellow. In card 2, patients read aloud the colour of the presented boxes.

##### Divided attention

Divided attention was assessed using subtest B of the TMT (Reitan, 1992). In subtest B, patients not only have to connect numbers, but also have to connect letters in ascending order, constantly shifting from number to letter.

##### Attention

Selective attention was assessed using card 3 of the SCWT (Stroop, 1935). In card 3, patients are requested to read aloud the ink of the words (red, green, blue, and yellow). Sustained attention was assessed using the d2 (Brickenkamp, Hängsen, Merten, & Hängsen, 2007). In this test, patients are asked to cancel out all ‘d’s’ with a total of two dashes either above or below the letter. There are a total of 14 rows, with a time limit of 20 s per row. The score on concentration performance (CP) was used for analyses, assessed as the number of correctly cancelled ‘d’s’ minus the incorrectly cancelled symbols (Brickenkamp *et al.*, 2007).

##### Executive functioning

Planning was assessed using the subtests of the Behavioral Assessment of the Dysexecutive Syndrome (BADS; Wilson, Alderman, Burgess, Emslie, & Evans, 1996): Key Search and Zoo Map. The Key Search subtest examines planning and strategy by asking patients to draw a route in a square on a piece of paper they would follow to find their lost key (Wilson *et al.*, 1996). The subtest Zoo Map examines complex planning and strategy by asking patients twice to draw a route they would follow to visit all animals presented on the list. In the first condition, patients are simply asked to visit the animals in the instruction. In the second condition, the order in which patients have to visit the animals is provided and patients have to follow that order (Wilson *et al.*, 1996).

Verbal fluency was measured using semantic and phonological verbal fluency tests (Deelman, Koning‐Haanstra, Liebrand, & Van der Burg, 1981). Patients are asked to name as much words as possible for one minute starting with a certain letter (N and A), followed by naming as much animals as possible for two minutes (Deelman *et al.*, 1981).

To assess cognitive flexibility, the Rule Shift Cards subtest of the BADS was used (Wilson *et al.*, 1996). The Rule Shift Cards subtest measures rule learning and rule shifting by asking patients to respond to a series of cards according to two presented rules.

##### Working memory

This domain was assessed using the WAIS‐IV Digit Span (Wechsler, 2008). The Digit Span subtest consists of three trials, in which patients are asked to verbally repeat given numbers in the same order, in reverse order, or in ascending order, respectively.

##### Memory

Verbal memory was assessed using the Rey Auditory Verbal Learning Test (RAVLT) and the Rivermead Behavioural Memory Test (RBMT). The RAVLT (Saan & Deelman, 1986) examines auditory–verbal memory by having patients remember and repeat 15 unrelated words, during a total of five trials. After 15–20 min, patients are asked to name all the words from that list they remembered (Saan & Deelman, 1986). The RBMT measures contextual memory by reading aloud two newspaper stories to patients (one at a time), after which they have to repeat the stories immediately, and repeat them again after 15 min (Wilson, Cockbum, & Baddeley, 1985).

Visual memory was assessed using the Rey–Osterrieth Complex Figure Test (ROCFT), immediate and delayed recall (Osterrieth, 1944). The test examines visual memory and visuospatial construction by asking patients to copy a complex figure, and subsequently reproduce the figure from memory. After 30 min, patients again have to reproduce the figure from memory. The total scores on these tests were converted into percentile scores.

##### Language

Language‐related functioning was assessed using the Boston Naming Test (BNT) and the verbal fluency test. The BNT examines confrontational word retrieval by having patients name or describe the presented pictures (Kaplan, Goodglass, & Weintraub, 2001).

##### Visuospatial construction

Visuospatial construction was assessed using the ROCFT (Osterrieth, 1944). Patients are asked to copy a complex figure, consisting on 18 identifiable areas. Those areas are scored with regard to the accuracy of the position, distortion, and/or absence of an area. A total score of 36 is the maximum score patients can achieve.

#### Demographic and clinical covariates

Demographic variables (age, sex, education) and clinical variables (depression, anxiety) were obtained during intake. The level of education was classified using the ‘Verhage coding scale’ (Verhage, 1964) and divided into low (Verhage 1–4), average (Verhage 5), and high (Verhage 6–7).

#### Depression and Anxiety assessment

Depression and anxiety were assessed during the stand PROM at intake. Depression was measured using the Patient Health Questionnaire‐9 (PHQ‐9; Kroenke, Spitzer, & Williams, 2001). This self‐report scale consists of nine items to measure depression severity. A cut‐off score of 10 was used to detect depression (Kroenke *et al.*, 2016), which had good psychometric properties (sensitivity and specificity of 88%). The Generalized Anxiety Disorder‐7 (GAD‐7; Spitzer, Kroenke, Williams, & Lowe, 2006) was used to measure anxiety. This self‐report questionnaire consists of seven items measuring the frequency of anxiety symptoms during the last two weeks. The psychometric properties of the GAD‐7 are good (Cronbach’s α = .8 to .9) (Spitzer *et al.*, 2006). A cut‐off score of 10 on the GAD‐7 was used to detect anxiety (Kroenke *et al.*, 2016). These questionnaires were obtained as part of routine outcome monitoring.

### Statistical methods

We examined descriptive statistics for demographic (e.g., age, sex, education level) and clinical variables (e.g., depression, anxiety) for the patients with CD/FND, patients with other SSRD and the total sample. Differences in demographic and clinical variables between the CD/FND and other SSRD patient groups were explored using chi‐square test and Cramer’s *V* or independent‐samples *t*‐test (and Cohen’s *d*) for categorical variables and continuous variables, respectively.

To test if the raw scores of the NPA tests were normally distributed, the Shapiro–Wilk test and Kolmogorov–Smirnov test were used in addition to visual inspection of the frequency histograms of each variable.

To compare patients with CD/FND with normative values, all raw scores were transformed into percentile scores by using the available norm scores. Patients were then classified as either having no neurocognitive problems (larger than or equal to the 20th percentile), having a deficit (between the 2.4th and 20th percentile) or having a disorder (smaller than the 2.4th percentile) (Lezak *et al.*, 2012).

To compare the CD/FND with the SSRD groups with respect to neurocognitive functioning, independent *t*‐tests (for normally distributed data) and the Mann–Whitney *U* test (for non‐normally distributed data) and the raw NPA test scores. Cohen’s *d* values are presented as index of effect size.

Multivariate analysis of variance (MANOVA) was conducted to examine whether depression or anxiety displayed an additional association with neurocognitive functioning in CD/FND. Because log transformations did not result in normal distribution, raw scores that deviated from normal were used for MANOVA. If an association for an overall MANOVA effect was observed, subsequent hierarchical multiple regression analyses were used to adjust for sex, age, education level, depression and anxiety (as categorical variables; analyses using continuous variables yielded the same results) with the neuropsychological tests that differed significantly between CD/FND and other SSRD as dependent variable. IBM Statistical Package for the Social Sciences Statistics 22.0 (IBM Corporation, 2013) was used for statistical analyses.

## Results

### Patient characteristics

Table 1 provides an overview of the patient characteristics, for both the total sample and differentiated by patients with CD/FND and those with other SSRD. Of the total sample of 318 patients, 61.9% were women, and patients had a mean age of 42.1 (*SD* = 13.3). A diagnosis of CD/FND was made in 29 (9.1%) patients, and 289 (90.9%) patients had other SSRD. Most of the patients were married, or had a registered partner, had an average level of education, and were not able to work due to physical complaints. 6.9% of the CD/FND group was suspected of malingering, compared to 7.3% of the other SSRD group. Further analyses were conducted using the total sample after the exclusion of patients who were suspected of malingering (*N* = 295).

**Table 1:** Descriptive statistics of total sample and differentiated by CD/FND and SSRD

	Total sample	CD/FND	Other SSRD	ES
	(*N* = 318)	(*N* = 29)	(*N* = 289)	
	*n* (%)/*M (SD)*	*n* (%)/*M (SD)*	*n* (%)/*M (SD)*	
Age (years)	42.1 (13.3)	42.4 (13.8)	42.1 (13.3)	−.02
Sex				
Woman	197 (61.9%)	23 (79.3%)	174 (60.2%)	.11*
Education level^a^				
Low (Verhage 1–4)	83 (26.1%)	11 (37.9%)	72 (24.9%)	.13
Average (Verhage 5)	131 (41.2%)	14 (48.3%)	117 (40.5%)	
High (Verhage 6–7)	98 (30.8%)	4 (13.8%)	94 (32.5%)	
Missing	6 (1.9%)	0 (0.0%)	6 (2.1%)	
Marital status				
Married/Registered partnership	123 (38.7%)	13(44.8%)	110 (38.1%)	.07
Partner	78 (24.5%)	7 (24.1%)	71 (24.6%)	
Single	88 (27.7%)	7 (24.1%)	81 (28.0%)	
Living with parents	6 (1.9%)	1 (3.4%)	5 (1.7%)	
Missing	23 (7.2%)	1 (3.4%)	22 (7.6%)	
Work status				
Full‐time/Part‐time	60 (18.9%)	2 (6.9%)	58 (20.1%)	.14
Unemployed/Retired	66 (20.8%)	9 (31.0%)	57 (19.7%)	
Cannot work due to physical complaints	117 (36.8%)	13 (44.8%)	104 (36.0%)	
Studying	6 (1.9%)	1 (3.4%)	5 (1.7%)	
Different/Unknown	69 (21.7%)	4 (13.8%)	65 (22.5%)	
Malingering				
Yes	23 (7.2%)	2 (6.9%)	21 (7.3%)	.09
No	256 (80.5%)	26 (89.7%)	230 (79.6%)	
No malingering tests assessed	39 (12.3%)	1 (3.4%)	38 (13.1%)	
Psychological measures				
Depressive symptoms (PHQ‐9)				
Mean score PHQ‐9	14.3 (6.1)	14.4 (6.5)	14.3 (6.0)	.02
Missing	2			
Positive for depression	242 (76.1%)	22 (75.9%)	220 (76.1%)	.01
No depression	74 (23.3%)	7 (24.1%)	67 (23.2%)	
No PHQ‐9 assessed	2 (0.6%)	–	2 (0.7%)	
Anxiety (GAD‐7)				
Mean score GAD‐7	11.6 (5.8)	10.1 (5.8)	11.8 (5.8)	.29
Missing	2			
Positive for Anxiety	197 (61.9%)	16 (55.2%)	181 (62.6%)	.05
No anxiety	119 (37.4%)	13 (44.8%)	106 (36.7%)	
No GAD‐7 assessed	2 (0.6%)	–	2 (0.7%)	

Notes

GAD = General Anxiety Disorder; PHQ = Patient Health Questionnaire.

Total number of patients per group (*N*), number of patients per variable (*n*) with percentages (%), and mean scores (*M*) with standard deviations (*SD*) per group are presented. Cramer’s *V* (chi‐square) and Cohen’s *d* (independent‐samples *t*‐test) were used to determine the effect size (ES).

^a^ Following Verhage coding (57).

* *p* < .05 (two‐tailed).

A total of 29 patients were diagnosed with CD/FND of which 6 patients presented with psychogenic non‐epileptic attacks, seven patients suffered from periods of unresponsiveness (including loss of speech) and 16 patients showed either weakness/paralysis or loss of sensation in extremities. These symptoms were evaluated previously in all patients with either EEG, CT scan,or MRI scan at a neurology department and/or an academic centre of epileptology. Regarding the 289 patients with other SSRD, two patients were diagnosed with factitious disorder, two with unspecified psychological disorder by a somatic disease, 23 patients were diagnosed with an illness anxiety disorder, and 262 patients were diagnosed with a somatic symptom disorder.

Sex differed significantly between patients with CD/FND and other SSRD (*d* = .11, *p* = .043). Mean scores on the PHQ‐9 and GAD‐7 did not differ significantly between patients with CD/FND and other SSRD.

### Neurocognitive functioning in patients with conversion disorder

Table 2 provides an overview of neurocognitive functioning for each cognitive domain in patients with CD/FND. Compared to population‐based normative reference data, combined deficits/disorders (i.e., scoring below the 20% percentile compared to normative values) were found in the domains information processing speed (Digit Symbol Substitution Test: 65.4%, TMT‐A: 55.5%, SCWT card 1: 88.5%, and SCWT card 2: 76.0%), divided attention (TMT‐B: 36.0%), selective attention (SCWT card 3: 12.0%), sustained attention (d2: 52.2%), and working memory (WAIS Digit Span: 51.8%). Patients with CD/FND also had impaired verbal memory based on the RALVT immediate (37.0%) and delayed (40.7%) recall, and the RBMT Story Recall immediate (23.1%) and delayed (26.9%) recall. Deficits/disorders were also found in visual memory according to the ROCFT immediate and delayed recall (50.0% and 53.9%, respectively). 40.0% of the patients had deficits/disorders within language (BNT). Executive functioning tests also displayed deficits/disorders including problems with planning based on the BADS Key Search (22.2%) and BADS Zoo Map (7.7%), as well as phonological verbal fluency (47.8%), and semantic verbal fluency (16.0%). Visuospatial construction (ROCFT copy: 100.0%) and cognitive flexibility (BADS Rule Shift Cards: 15.4%) were also impaired.

**Table 2:** Neurocognitive functioning of patients with CD/FND (*N* = 29)

Neurocognitive domain	Raw scores	Percentiles
	*M (SD)*	*n* (%)
Information processing speed		
WAIS Digit Symbol Substitution Test (*N* = 26)		
No neurocognitive problems	57.2 (19.1)	9 (34.6%)
Deficit		11 (42.3%)
Disorder		6 (23.1%)
TMT‐A (*N* = 27)		
No neurocognitive problems	43.2 (17.5)	12 (44.4%)
Deficit		10 (37.0%)
Disorder		5 (18.5%)
SCWT Card 1 (*N = *26)		
No neurocognitive problems	62.0 (15.1)	3 (11.5%)
Deficit		10 (38.5%)
Disorder		13 (50.0%)
SCWT Card 2 (*N = 25)*		
No neurocognitive problems	79.6 (24.6)	6 (24.0%)
Deficit		4 (16.0%)
Disorder		15 (60.0%)
Attention		
Divided attention: TMT‐B (*N* = 25)		
No neurocognitive problems	104.6 (60.2)	16 (64.0%)
Deficit		4 (16.0%)
Disorder		5 (20.0%)
Selective attention: SCWT Card 3 (*N* = 25)		
No neurocognitive problems	124.8 (61.8)	22 (88.0%)
Deficit		3 (12.0%)
Disorder		
Sustained attention: d2 (*N* = 23)		
No neurocognitive problems	132.5 (46.1)	11 (47.8%)
Deficit		10 (43.5%)
Disorder		2 (8.7%)
Executive functioning		
Planning		
BADS Key Search *(N* = 27)		
No neurocognitive problems	11.9 (4.3)	21 (77.8%)
Deficit		1 (3.7%)
Disorder		5 (18.5%)
BADS Zoo Map *(N* = 26)		
No neurocognitive problems	11.5 (4.5)	24 (92.3%)
Deficit		2 (7.7%)
Disorder		–
Verbal fluency		
Phonological verbal fluency: N + A (*N* = 23)		
No neurocognitive problems	18.2 (7.8)	12 (52.2%)
Deficit		11 (47.8%)
Disorder		–
Semantic verbal fluency: animals (*N* = 25)		
No neurocognitive problems	30.2 (8.1)	21 (84.0%)
Deficit		3 (12.0%)
Disorder		1 (4.0%)
Memory processes		
Working memory: WAIS Digit Span (*N* = 27)		
No neurocognitive problems	22.2 (5.9)	13 (48.1%)
Deficit		8 (29.6%)
Disorder		6 (22.2%)
Storage of information		
Verbal Memory: RALVT immediate recall *(N* = 27)		
No neurocognitive problems	40.2 (11.7)	17 (63.0%)
Deficit		3 (11.1%)
Disorder		7 (25.9%)
Verbal Memory: RBMT immediate recall *(N* = 26)		
No neurocognitive problems	16.4 (6.1)	20 (76.9%)
Deficit		6 (23.1%)
Disorder		–
Retrieval of information		
Verbal Memory: RALVT delayed recall (*N* = 27)		
No neurocognitive problems	8.1 (3.7)	16 (59.3%)
Deficit		6 (22.2%)
Disorder		5 (18.5%)
Verbal Memory: RBMT delayed recall (*N* = 26)		
No neurocognitive problems	12.7 (6.0)	19 (73.1%)
Deficit		5 (19.2%)
Disorder		2 (7.7%)
Visual Memory: ROCFT immediate recall *(N* = 26)		
No neurocognitive problems	17.9 (7.7)	13 (50%)
Deficit		9 (34.6%)
Disorder		4 (15.4%)
Visual Memory: ROCFT delayed recall (*N* = 26)		
No neurocognitive problems	16.0 (7.3)	12 (46.2%)
Deficit		6 (23.1%)
Disorder		8 (30.8%)
Language		
Word retrieval: BNT*(N* = 25)		
No neurocognitive problems	159.0 (13.4)	15 (60.0%)
Deficit		7 (28.0%)
Disorder		3 (12.0%)
Visuospatial construction		
ROCFT copy *(N* = 26)		
No neurocognitive problems	28.6 (6.4)	–
Deficit		18 (69.2%)
Disorder		8 (30.8%)

Notes

BADS, Behavioral Assessment of the Dysexecutive Syndrome; BNT, Boston Naming Test; RAVLT, Rey Auditory Verbal Learning Test; RBMT, Rivermead Behavioural Memory Test; ROCFT, Rey–Osterrieth Complex Figure Test; ROCFT, Rey–Osterrieth Complex Figure Test; SCWT, Stroop Color–Word Test; TMT, Trail Making Test; WAIS, Wechsler Adult Intelligence Scale.

Total number of patients per group (*N*), and number of patients per variable (*n*) with percentages (%) are presented. Deficit = ≤20th percentile compared to normative values (but >2.4 percentile), disorder = ≤2.4 percentile compared to normative data.

### Neurocognitive functioning in patients with conversion disorder versus patients with other SSRD

Neurocognitive functioning of patients with CD/FND compared to patients with other SSRD is described in Table 3. Patients with SSRD other than CD/FND displayed deficits in 14 out of the 21 neurocognitive tests. When comparing patients with CD/FND with patients with other SSRD using non‐parametric Mann–Whitney (U) tests (on the raw scores), we found that patients with CD/FND performed significantly worse on the TMT‐A (*U* = 2,579.5, *z* = −2.243, *p* = .025, *d* = −.31), SCWT card 1 (*U* = 1,816.5, *z* = −3.872, *p* < .001, *d* = −.82), card 2 (*U* = 1,928.5, *z* = −3.339, *p* = .001, *d* = −.88), and card 3 (*U* = 2,375.5, *z* = −2.175, *p* = .030, *d* = −.54), the ROCFT copy (*U* = 2,527.0, *z* = −2.031, *p* = .042, *d* = .44), and the phonological part of the verbal fluency test (*U* = 2,382.5, *z* = −2.010, *p* = .044, *d* = .45).

**Table 3:** Neurocognitive functioning of patients with CD/FND compared to other SSRD

	Raw scores *M (SD)*		
	CD/FND	Other SSRD	Cohen’s *d*
Information processing speed			
WAIS Digit Symbol Substitution Test	57.2 (19.1)	64.2 (17.6)	.39
*(N* ^a^)	26	251	
TMT‐A	43.2 (17.5)	37.3 (18.9)	−.31*
*(N* ^a^)	27	259	
SCWT Card 1	62.0 (15.1)	50.6 (13.8)	−.82***
*(N* ^a^)	26	259	
SCWT Card 2	79.6 (24.6)	63.9 (17.1)	−.88**
*(N* ^a^)	25	259	
Attention			
Divided attention: TMT‐B	104.6 (60.2)	82.5 (47.6)	−.45
*(N* ^a^)	25	257	
Selective attention: SCWT Card 3	124.8 (61.8)	102.4 (39.0)	−.54*
*(N* ^a^)	25	258	
Sustained attention: d2	132.5 (46.1)	144.3 (46.1)	.26
*(N* ^a^)	23	250	
Working memory			
WAIS Digit Span	22.2 (5.9)	24.4 (5.2)	.42
*(N* ^a^)	27	261	
Storage of information (memory)			
Verbal Memory: RALVT immediate recall	40.2 (11.7)	42.2 (10.8)	.18
*(N* ^a^)	27	261	
Verbal Memory: RBMT Story immediate recall	16.4 (6.1)	17.1 (6.1)	.11
*(N* ^a^)	26	260	
Retrieval of information			
Verbal Memory: RALVT delayed recall	8.1 (3.7)	8.6 (3.1)	.06
*(N* ^a^)	27	262	
Verbal Memory: RBMT Story delayed recall	12.7 (6.0)	13.9 (5.8)	.21
*(N* ^a^)	26	256	
Visual Memory: ROCFT immediate recall	17.9 (7.7)	18.9 (7.0)	.14
*(N* ^a^)	26	253	
Visual Memory: ROCFT delayed recall	16.0 (7.3)	18.5 (6.8)	.37
*(N* ^a^)	26	252	
Language			
Word retrieval: BNT	159.0 (13.4)	157.8 (16.3)	−.07
*(N* ^a^)	25	260	
Visuospatial construction			
ROCFT copy	28.6 (6.4)	31.0 (5.3)	.44*
*(N* ^a^)	26	256	
Executive functioning			
Phonological verbal fluency: N + A	18.2 (7.8)	22.3 (9.2)	.45*
*(N* ^a^)	25	252	
Semantic verbal fluency: animal naming	30.2 (8.1)	32.4 (8.9)	.25
*(N* ^a^)	25	252	
Cognitive flexibility: BADS Rule Shift Cards	18.7 (2.7)	19.0 (2.4)	.12
*(N* ^a^)	26	252	
Planning			
BADS Key Search	11.9 (4.3)	11.9 (3.8)	.00
*(N* ^a^)	27	255	
BADS Zoo Map	11.5 (4.5)	11.6 (4.0)	.02
*(N* ^a^)	26	250	

Notes

BADS = Behavioral Assessment of the Dysexecutive Syndrome; BNT = Boston Naming Test; RAVLT = Rey Auditory Verbal Learning Test; RBMT = Rivermead Behavioural Memory Test; ROCFT = Rey–Osterrieth Complex Figure Test; SCWT = Stroop Color–Word Test; TMT = Trail Making Test; WAIS = Wechsler Adult Intelligence Scale.

Mean scores (*M*) with standard deviations (*SD*), and number of patients per variable (*n*). Because all planning tasks were not normally distributed, analyses were conducted using the Mann–Whitney *U* test. Cohen’s *d* was used to determine the effect size (ES).

^a^ Total number of patients per group.

* *p* < .05

** *p* < .01

*** *p* < .001 (two‐tailed).

Analysis of potentially confounding variables revealed that depression was not significantly associated with the neurocognitive measures that were different between patients with CD/FND versus those with other SSRD (Wilks Lamba (*F*(21)= .901, *p* = .591, partial eta squared = .083). Anxiety was also not significantly related to neurocognitive measures that were different between patients with CD/FND versus those with other SSRD, Wilks Lamba (*F*(21) = .765, *p* = .761, partial eta squared = .071. Therefore, no further multiple regression analyses were conducted to explore the additional effect of depression and anxiety on the neurocognitive domains in patients with CD/FND.

## Discussion

This study shows that patients with CD/FND show substantial neurocognitive impairments (i.e., disorders and deficits combined) compared to population level, within the domains of information processing speed, attention, executive functioning, (working) memory, and language. No impairments were found in visuospatial functioning in patients with CD/FND, which is in contrast with our a priori expectations. Compared to patients with other SSRD, patients with CD/FND showed significantly more impairment in information processing speed. This finding was in line with our hypothesis. Depression and anxiety, or education, age, and gender, did not play an important role in the differences in neurocognitive functioning in patients with CD/FND versus those with other SSRD.

When comparing the results of this study to the available literature, neurocognitive impairments in the domains attention, working memory, verbal and visual memory, visuospatial functioning, and information processing speed are commonly found in patients with CD/FND (Brown *et al.*, 2014; Demir *et al.*, 2013; Kozlowska *et al.*, 2015). This study also adds dysfunction in the language domain to the list of impairments, which are also found in the other SSRD group (Al‐Adawi *et al.*, 2010; Grace *et al.*, 1999; Hall *et al.*, 2011; Hart *et al.*, 2000; Michiels & Cluydts, 2001; Moore *et al.*, 2012; Niemi *et al.*, 2002). Our results show that patients with CD/FND have substantially impaired information processing speed which may also influence other neurocognitive functions (Penke, Maniega, Bastin, Valdés Hernández, Murray, Royle, *et al.*, 2012; Verhaeghen, 2014).

Regarding the neurobiological mechanisms that could play a role in these associations, evidence suggests that brain areas that are linked to CD/FND include the supplementary motor area, dorsolateral prefrontal cortex (dlPFC), and anterior cingulate cortex (Ridderinkhof, Van den Wildenberg, Segalowitz, & Carter, 2004; Rubia, Russell, Overmeyer, Brammer, Bullmore, & Sharma, 2001). In particular, the anterior cingulate cortex is important for the cognitive and attentional aspect of the inhibitory responses as measured using the Stroop Color–Word task (Rubia *et al.*, 2001). The dlPFC is involved in the decision‐making process and selection of the responses by linking the representation of short‐term memory with goal‐directed motor behaviour (Ridderinkhof *et al.*, 2004). The cerebellum is one of the brain areas involved in inhibition of motor responses (Rubia *et al.*, 2001). However, the cerebellum is also an important brain area for other cognitive functions, including attention, executive functioning, (working) memory, language, and visuospatial regulation (Baillieux, De Smet, Paquier, De Deyn, & Marien, 2008). Thus, there is a plausible interplay between motor and sensory symptoms of CD/FND, the neurobiological correlates of CD/FND and neuropsychological functioning in the domains of attention, inhibition, learning, (working) memory, executive functioning, and visuospatial functioning. Evidence from this study suggests that basic information processing speed may be disproportionately impaired in CD/FND versus other SSRD, which may in part reflect supplementary motor areas as well as cerebellum dysfunction.

The findings of this study have implications for the treatment of CD/FND. In general, the effectiveness of CBT remains equivocal (Conwill, Oakley, Evans, & Cavanna, 2014; Goldstein *et al.*, 2010; Kuyk, Siffels, Bakvis, & Swinkels, 2008; LaFrance *et al.*, 2014) for treating psychogenic non‐epileptic seizures. A review of randomized controlled trials (Kroenke, 2007) reported only two randomized controlled trials (RCT) which explored the effect of hypnosis in patients with CD but showed limited effects (Moene, Spinhoven, Hoogduin, & Van Dyck, 2002; Moene, Spinhoven, Hoogduin, & Van Dyck, 2003 ). Other therapies, so‐called ‘third wave’ therapies, in which mindfulness is incorporated as psychotherapeutic elements are gaining evidence as effective interventions (Baslet & Hill, 2011; Carlson & Perry, 2017). Concluding, evidence‐based treatments for CD/FND are scarce and require further studies.

Nevertheless, the effect of therapy may be limited in cases in which patients with CD/FND also experience problems with information processing speed. For instance, CBT sessions are harder to follow, and the information consolidation during the treatment session is limited due to impaired information processing speed which also limits the information retrieval after the treatment session. In that sense, problems in neurocognitive functioning might negatively influence treatment outcomes. Focusing on neurocognitive problems by using a cognitive rehabilitation therapy (CRT) can provide a useful tool to support neurocognitive problems in CD/FND patients with cognitive impairment (De Vroege, Khasho, Foruz & Van der Feltz‐Cornelis, 2017) but also improve motor symptoms and mental disorders. As a result, the neurocognitive symptoms of patients with CD/FND can be of great burden and may play a pivotal role within treatment Nevertheless, neurocognitive problems may be overcome in a preliminary stage before the start of CBT by using CRT (Laatsch & Krisky, 2006).

Cognitive rehabilitation therapy is a treatment that is used successfully in patients with brain injury for a long time and is a standard approach for this patient population (Laatsch & Krisky, 2006). A study combining CRT with functional magnetic resonance imaging (fMRI) activity in traumatic brain injury patients reported both improvements in neurocognitive functioning and increased activity in almost all brain areas similar to healthy controls and activation of other brain areas after CRT (Laatsch & Krisky, 2006). These preliminary results show that CRT has great potential with regard to improvement of neurocognitive functioning and the activity of involved brain areas. A recent case study already showed that using CRT in a patient with CD/FND not only contributed to better neurocognitive functioning, but also improved motor symptoms and mental disorders (De Vroege *et al.*, 2017). However, more research on effectivity of CRT in patients with CD/FND has yet to be conducted. Future studies are needed to evaluate improvements in neurocognitive functioning after CRT and to establish the effect of CRT on CD/FND specific symptoms. Furthermore, the literature suggests a possible role of directing attention towards symptoms as an important determinant of CD/FND symptomatology (e.g., McIntosh, McWhirter, Ludwig, Carson, & Stone, 2017). Future studies are needed to empirically investigate the consequences of diverting attention towards or away from (neurocognitive) symptoms in patients with CD/FND.

### Limitations and strengths

Interpretation of the present study findings needs to be considered in the context of several limitations. The group of patients with CD/FND is relatively small compared to the group of patients with other SSRD. With regard to the neuropsychological battery, the population‐based normative reference data of the ROCFT copy only classified patients with a maximum of >16th percentile. This potentially leads to confounding results, given that patients can never be classified as having no neurocognitive problems and the percentage of patients with impairment in both groups is 100%. Another limitation is the use of data that did not follow the normal distribution to conduct the MANOVA. Moreover, potentially confounding factors such as medication use (e.g., oxycodone) (Cherrier, Amory, Ersek, Risler, & Shen, 2009) and comorbid disorders (e.g., posttraumatic stress disorder, attention deficit hyperactivity disorder) (Millan *et al.*, 2012), which could have had an effect on neurocognitive functioning, were not taken into account. These limitations are outweighed to some extent by several strengths of this study, including the unique focus on neurocognitive functioning in CD/FND using other SSRD patients as reference group, and the assessment of a large number of neurocognitive tests and broad range of cognitive domains relevant to CD/FND.

### Conclusions and recommendations for future research

This study documents impairments in neurocognitive functioning in patients with CD/FND and compared these results to neurocognitive functioning of other SSRD patients. We demonstrated that impairments in information processing speed may be disproportionately present in CD/FND versus other manifestations of SSRD, whereas other neurocognitive problems may be common to all SSRD, including CD/FND (e.g., attention, working memory, memory, language, visuospatial construction, and aspects of executive functioning such as cognitive flexibility and planning. Furthermore, neurocognitive dysfunctioning may lead to less efficient CBT, the treatment of choice in CD/FND. If CBT is negatively influenced by impaired cognitive functioning (i.e., patients forget the information that was taught during session or forget to do homework), CBT may not be the effective treatment that patients with CD/FND need. CRT, in order to overcome neurocognitive problems, prior to CBT may offer a solution.

Future research on neurocognitive functioning in CD/FND compared to other SSRD should include larger samples of patients with CD/FND. Comorbid disorders should also be included in the study to examine the direct association of CD/FND or other SSRD with impaired neurocognitive functioning. Furthermore, it is important to combine neurocognitive functioning in CD/FND with neurobiological correlates in future studies by using fMRI. Finally, to investigate whether CRT has positive effects on patients with CD/FND suffering from neurocognitive impairment, future studies are needed to explore the effect of CRT on mental and physical health outcomes in intervention studies targeting patients with CD/FND and other SSRD.

## Conflicts of interest

All other authors declare no conflict of interest.

## Author contributions

Lars de Vroege (Conceptualization; Formal analysis; Methodology; Writing – original draft; Writing – review & editing) Iris Koppenol (Data curation; Formal analysis; Writing – original draft; Writing – review & editing) Madelon M.E. Riem (Writing – original draft; Writing – review & editing) Willem Johan Kop (Conceptualization; Formal analysis; Methodology; Supervision; Writing – original draft; Writing – review & editing) Christina Maria van der Feltz‐Cornelis (Conceptualization; Formal analysis; Methodology; Supervision; Writing – original draft; Writing – review & editing).

## Data Availability

Data available on request due to privacy/ethical restrictions.
